# A quantitative method for determination of colistin E2 methanesulphonate in human plasma by ^15^N-labeled colistin E2

**DOI:** 10.1038/s41598-023-45256-3

**Published:** 2023-10-26

**Authors:** Hongjiang Xu, Yanan Li, Jing Zhang, Jinhua Zhang, Jianguang Lu, Xiquan Zhang, Ling Yang, Wenjie Zhao, Jun Feng

**Affiliations:** 1 China State Institute of Pharmaceutical Industry, Shanghai, China; 2 Chia Tai Tianqing Pharmaceutical Group Co., Ltd., Nanjing, China; 3 Department of Biological Medicines and Shanghai Engineering Research Centre of Immuno-Therapeutics, School of Pharmacy, Fudan University, Shanghai, China; 4 Institute of Antibiotics, Huashan Hospital, Fudan University, Shanghai, China; 5 Shanghai Duomirui Biotechnology Ltd, Shanghai, China

**Keywords:** Analytical biochemistry, Biophysical chemistry

## Abstract

The single-component colistin E2, with superior antibacterial activity and lower toxicity, was being developed as the latest generation of polymyxin drugs. However, colistin E2 has not been tested quantitatively in biological matrices. In this study, based on the quantitative detection of colistin methanesulphonate (CMS) and colistin by Zhao et al., ^15^N-labeled colistin E2 was used as an internal standard (IS) for a more accurate quantitative detection of CMS E2 in human plasma. A rapid ultra-high-performance liquid chromatography-tandem mass spectrometry (UHPLC–MS/MS) assay method was developed for determination of CMS E2 and colistin E2 in human plasma. After pretreatment of plasma samples by 96-well SPE Supra-Clean Weak Cation Exchange (WCX) plate, the formed colistin E2 was detected and quantified by UHPLC–MS/MS system. All plasma lots were found to be free of interferences with the analyte. The matrix has no effect on the quantitation of the analyte. No significant effect of the carryover was observed. The dilution integrity was demonstrated in plasma samples without the loss of accuracy and precision. The lower limit of quantification (LLOQ) was 0.0300 mg/L for colistin E2 in plasma with accuracy (relative error, 5.1–12.7%) and precision (relative standard deviation,  − 5.7–9.3%). Stability of CMS E2 and colistin E2 was demonstrated in biological samples before and during sample treatment, and in the extract. Furthermore, this method was successfully applied to the analysis of plasma samples obtained from Chinese healthy volunteers receiving a single intravenous CMS E2 dose of 5 mg/kg. In conclusion, the detection method was characterized by speed and high accuracy, which laid a solid foundation for the subsequent development of CMS E2 drug.

## Introduction

Multidrug-resistant (MDR) Gram-negative bacteria represent a serious and growing risk to public health^1,2^. To date, the bacterial natural product polymyxin has been used as the last line of defense against serious infections caused by a number of MDR Gram-negative pathogens, especially carbapenem-resistant ones. Polymyxins are cyclic lipopeptide antibiotics produced by *Paenibacillus colistinus* that contain a variety of components^3^. Polymyxin B and colistin are two types of polymyxin drugs that are used clinically. Among these, colistin has been widely used because of its higher safety and fewer toxic side effects. Colistin is commercially available in two forms: colistin sulfate and colistin methane sulfonate (CMS), which are inactive prodrugs of colistin^4^. CMS is superior to colistin sulfate owing to its lower renal toxicity^5^.

**Figure 1:**
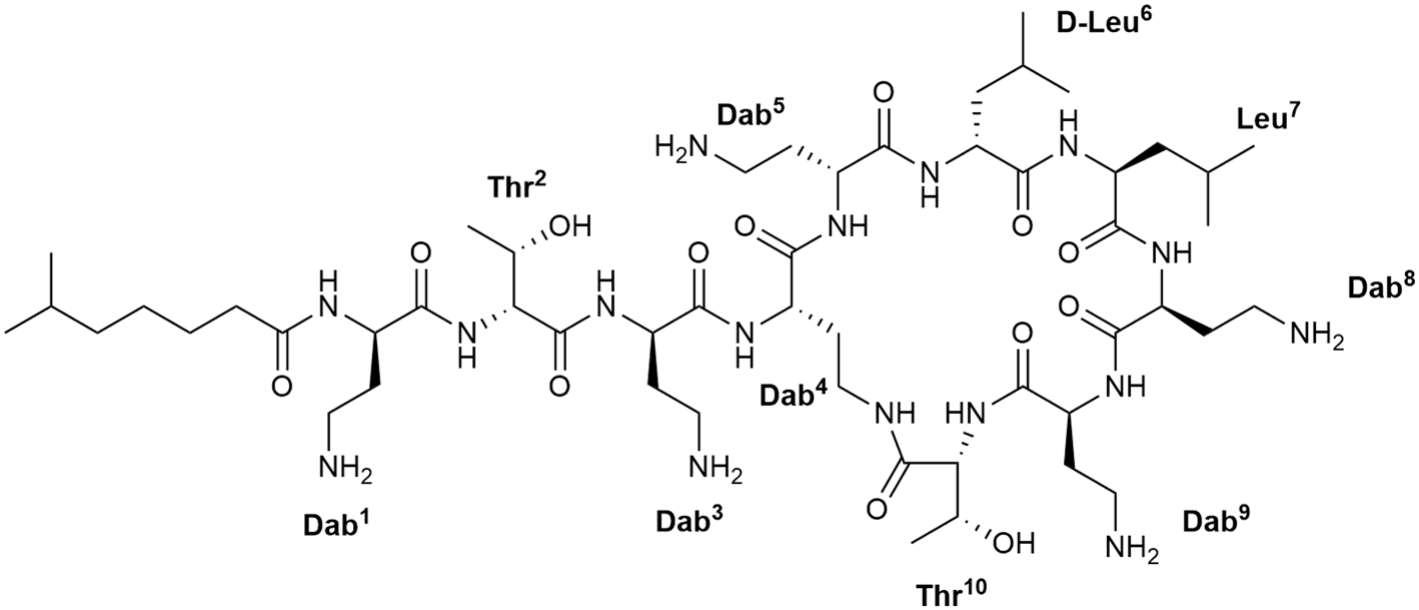
Chemical structure of colistin E2.

Notably, colistin is a complex mixture consisting of closely related compounds. In addition to at least 30 minor polymyxins with very similar physiochemical properties, colistin consists of colistin E1 and colistin E2 (Fig. 1)^6^. According to European Pharmacopoeia records, the components with the same molecular weight as colistin E1 are colistin E1-7MOA and E1-I, and colistin E2 is colistin E3, E2-I, and E1-Nva. In short, except for the principal components colistin E1 and E2, other components with similar physicochemical properties account for nearly 20% of colistin. These components could not be separated from colistin E1/E2 using mobile phases containing formic acid but were identified as a single peak of polymyxin E1/E2 by HPLC. This neglected problem actually brings some interferes with single component or colistin quantification. In the process of developing CMS, we successfully purified single components of colistin E1 and E2 with a purity of more than 99% by preparative HPLC using a specific salt mobile phase. Unexpectedly, the antibacterial activity of colistin E2 was found to be higher than that of E1. Based on the cylinder-plate method in the United States pharmacopoeia, we used *Bordetella bronchitica* ATCC4617 as the test bacteria and determined that the potency of CMS E1 was 319 µg/mg and that of CMS E2 was 611 µg/mg, which was nearly double that of CMS E1. Therefore, we developed colistin E2, which has superior antibacterial activity and lower toxicity, as the latest generation of polymyxin drug.

To accurately quantify in vivo pharmacokinetics of CMS E2, speed up its listing, and benefit clinical patients, it is critical to develop a method for more accurate quantitative detection of CMS E2 in human plasma. In recent years, several methods, such as weak cationic solid phase extraction, hydrophilic-lipophilic-balanced solid phase extraction, post-column derivatization, and in-line molecularly imprinted solid-phase extraction combined with high-performance liquid chromatography-tandem mass spectrometry (HPLC–MS/MS), have been used for the determination of colistin and CMS in biological samples^7–11^. However, the ISs used in the above studies were analogs of the subject to test (polymyxin B1/B2) or polymyxin B (a mixture). Notably, the analytical conditions of formic acid used could not distinguish colistin A/B from other components with similar physiochemical properties; therefore, there was a certain bias when polymyxin B/B1/B2 was used as the IS to quantify colistin in various biological matrices. In our study, ^15^N-labeled single-component colistin E2 with purity > 99% was used as an IS to quantitatively determine CMS E2 in human plasma by UHPLC-MS/MS, which is different from other studies and ensures a more accurate analysis.

## Materials and methods

### Chemicals

Colistin E2 and CMS E2 were provided by Chia Tai Tianqing Pharmaceutical Co., Ltd. ^15^N-labeled colistin E2 (which has the same structure as colistin E2; however, its 16 N atoms are ^15^N) was obtained from Shanghai Domirui Biotechnology Co., Ltd. Ultra-purified water was obtained using a Millipore Milli-Q water purification system (Millipore, Brussels, Belgium). HPLC-grade methanol, acetonitrile (ACN) and isopropanol were purchased from Merck. ACS grade 30% ammonia was purchased from Sigma. MS-grade formic acid (FA) was obtained from ACS. Oasis WCX 96-well plates (30 mg/1 ml), collection plates, and mats were obtained from Waters (Milford, MA, USA). Blank human plasma and whole blood (EDTA-K2 anticoagulation) were obtained from Chia Tai Tianqing Pharmaceutical Co., Ltd.

### Instrumental conditions

A Sciex Exion LC system (Sciex, USA) and Phonomenex Kinetex XB-C18 (50*2.1 mm, 2.6 μm, 10 nm) were used. The mobile phase consisted of solution A (water with 0.1% FA), solution B (ACN: methanol, 1:1), needle wash 01 (port, methanol: ACN: isopropanol: water: FA, 3:3:3:1:0.2) and needle wash 02 (pump, water containing 10% isopropanol). Using a flow rate of 0.4 mL/min at 40℃, the elution gradients are presented in Table 1. The total run time was 5 min and the injection volume was 2 μl. The UHPLC was interfaced to a Sciex Triple quad 5500 mass spectrometer (Sciex, USA), which monitored the precursor and product ions transition using the MRM scan mode (*m/z* 386.2/101.1 for colistin E2 and 391.4/103.1 for IS).

**Table 1:** List of elution conditions for UHPLC-MS/MS.

Time (min)	0	0.4	2.0	2.1	2.3	3.1	3.2	5.0
%B	15	15	15	90	90	90	15	end
Switching valve(position)		MS			Waste liquids			

### HPLC–UV for colistin E2 purity determination

According to the HPLC method for determination of colistin E2 purities (Chinese Pharmacopoeia 2020 edition of the four general principles 0512), YMC Pro Pack C18 (4.6*250 mm, 3 μm) was used; sodium sulfate solution (4.46 g of anhydrous sodium sulfate, 900 ml of water, the pH value to 2.4, phosphoric acid, and fixed volume to 1000 ml with water): ACN (78:22, v/v) was used as the mobile phase; the detection wavelength was 215 nm; the flow rate was 1.0 ml/min; the total run time was 35 min; the column temperature was 30°C.

### Standard solutions

The nominal concentration ranges of the calibration standards were in the range of 0.03–30 mg/L for colistin E2, whereas the QCs were 0.09, 0.24, 12, 24, and 120 mg/L for colistin E2. The final concentrations are expressed as mg/L colistin E2 as the free base. For conversion into molar units, molecular weights of 1155.46 for colistin E2 and 1170.70 for IS were used.

### Sample pretreatment and calculations

Each plasma sample was divided into two aliquots. The first aliquot was used to determine of free colistin E2 levels, and the second aliquot was used to quantify CMS E2 levels.

#### Determination of formed colistin E2

Referring to Zhou et al.^10^, for each plasma sample, 100 µL of 5% ammonia was added to a mixture containing 100 µL of CMS E2 calibration plasma standards or QCs and 20 µL of 15 µg/ml IS solution. After vortexing, the mixtures were loaded onto WCX SPE plates. Before loading the sample, the WCX SPE plates were preconditioned with 1 ml methanol followed by 1 mL water to activate the extraction. The plate was sequentially washed with 1 mL of water, 1 mL of methanol, 1 mL of 70% ACN with 2% ammonia, and 1 ml 0.5% FA. Subsequently, 300 µL of 70% ACN with 6% FA was used to elute the analytes twice. Total eluents (600 µL) were collected with an SPE 96-Deep Square Well collection plate, mixed in a 96-well plate mixer, and analyzed by UHPLC-MS/MS.

#### Determination of CMS E2

A volume of 1 mol/L sulfuric acid (50 μl) was mixed with 100 μl plasma, 2 mol/L NaOH (50 μl) was added after 15 min, vortexed for 3 min, and 200 µL of water was added. Following conversion to colistin E2, the concentrations of colistin E2 were determined using the methodology described above. The concentration of CMS E2 was calculated as C_2_ = (C_total_ − C_1_) × MW_CMS E2_/ MW_E2_, where C_2_ is the concentration of CMS E2, C_total_ is the total colistin E2 concentration in human plasma, including the free colistin E2 and colistin E2 converted from CMS E2, C_1_ is the concentration of the free colistin E2, MW_E2_ (1155) is the molecular weight of colistin E2, and MW_CMS E2_ (1735) is the average molecular weight of CMS E2.

### Validation methodology

The UHPLC-MS/MS method for the determination of colistin E2 concentrations was validated with the Bioanalytical Method Validation Guidance for Industry by the Food and Drug Administration (FDA)^12^ and the Guideline on Bioanalytical Method Validation by the European Medicines Agency (EMA)^13^.

### Statements

The clinical trial study was conducted in accordance with the ethics and scientific principles stipulated in the Helsinki Declaration and the Chinese Good Clinical Practice (GCP).

## Results and discussion

### Purity of colistin E2

**Figure 2:**
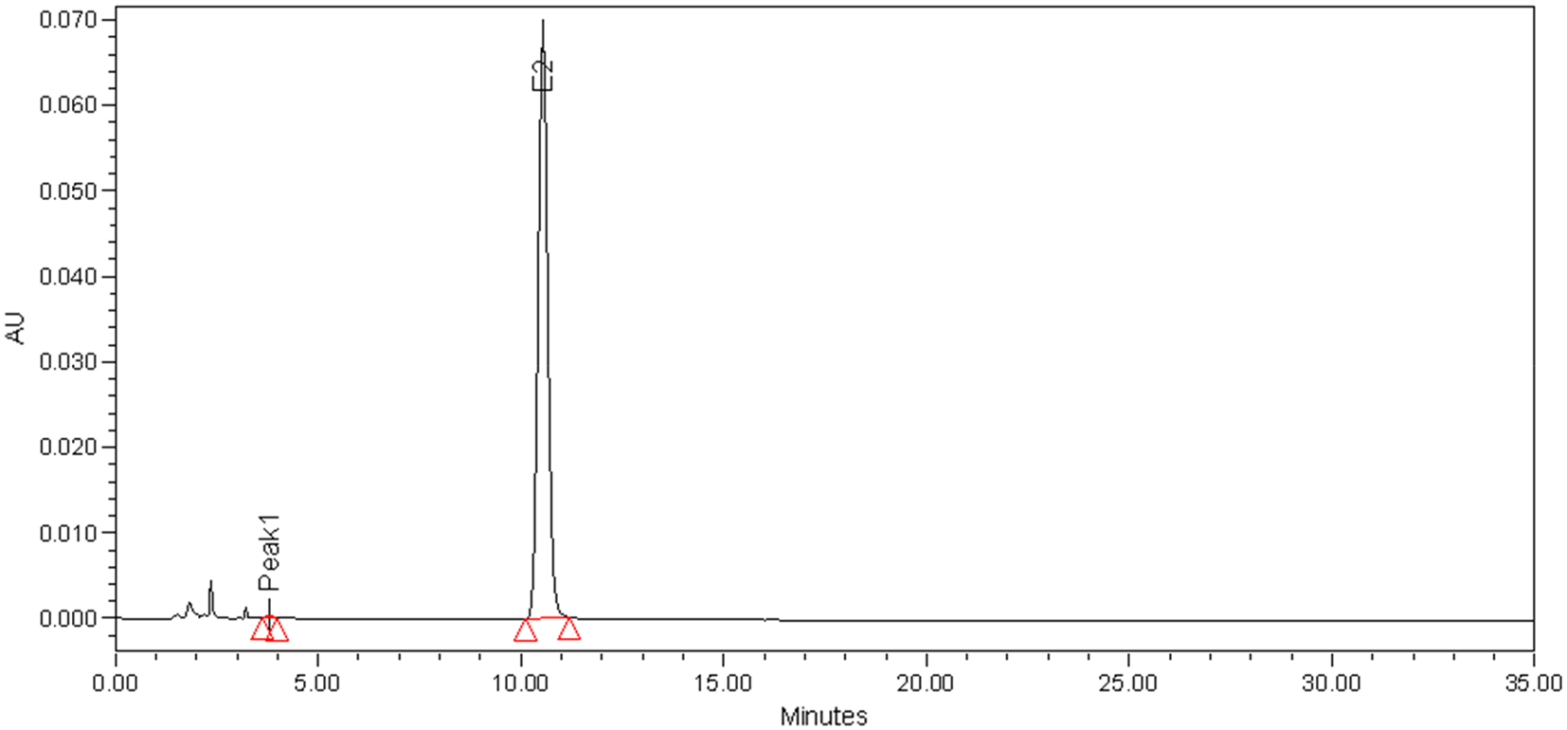
HPLC peak of colistin E2.

In our previous study, we isolated and purified colistin E2 with a purity of more than 99% from colistin using a specific salt mobile phase. The relative signal response value for colistin E2 determined by HPLC–UV analysis was 99.75% (Fig. 2). The peak eluted at 11.922 min was attributed to colistin E2 (consistent with the retention time of IS; data not shown).

### UHPLC-MS/MS method development

To prevent the presence of interference peaks and make quantification more accurate, ^15^N-labeled colistin E2 was used as the IS instead of polymyxin B mixtures^14,15^ or purified polymyxin B1^10^. An SPE WCX column-based sample preparation method was used to prepare plasma samples^10^. Before being loaded onto WCX SPE plates, the plasma samples were alkalized with 5% ammonia to make CMS E2 negatively charged and colistin E2 positively charged. Therefore, colistin E2 was retained, whereas CMS E2 was removed during the initial washing steps. Washing away unstable CMS E2 is critical for the accurate quantification of free colistin E2 in human plasma.

### Validation

#### Selectivity

**Figure 3:**
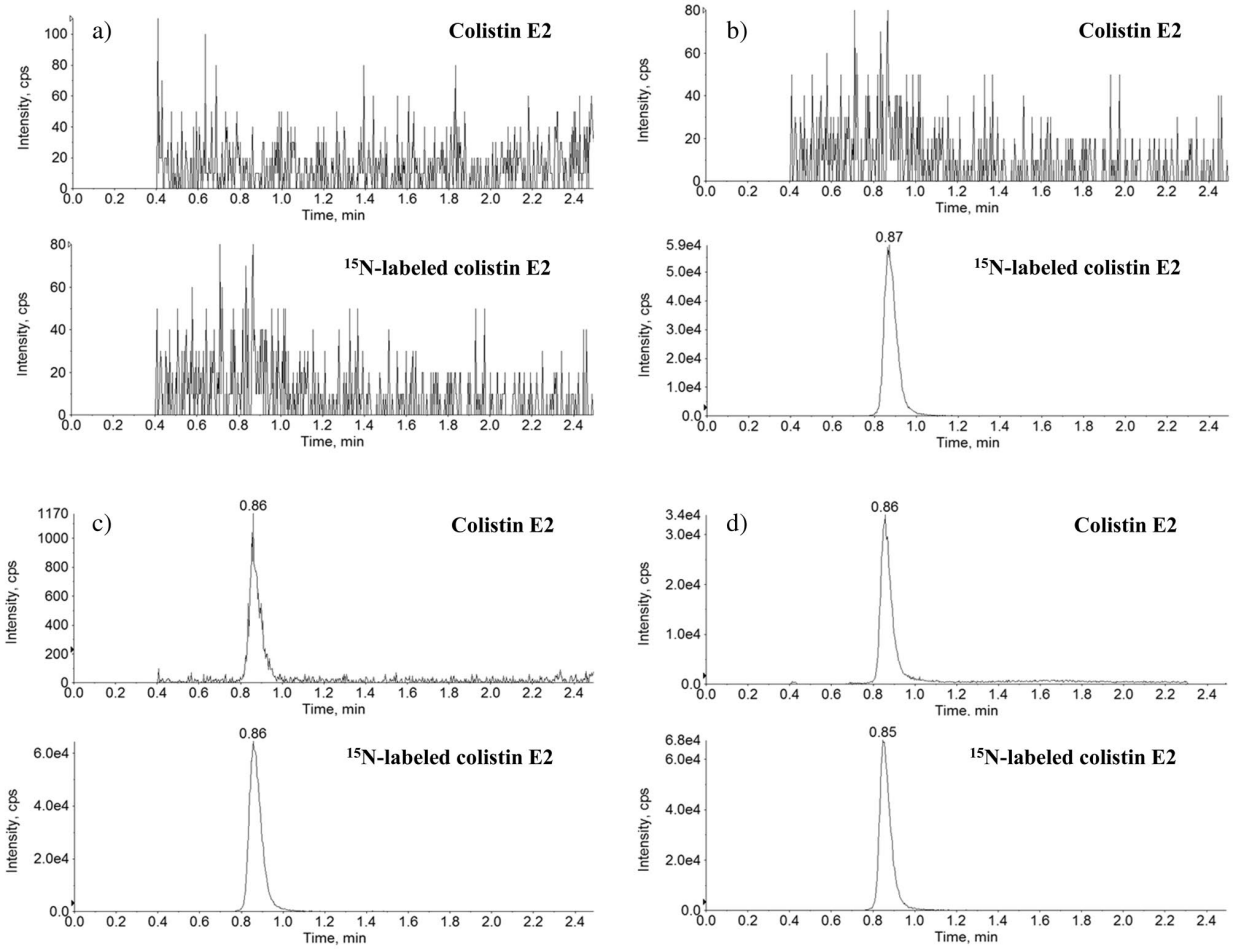
Typical UHPLC-MS/MS chromatograms of colistin E2 and IS in human plasma (n = 6): a) Blank plasma sample; b) Spiked human plasma sample with IS at 15 mg/L; c) spiked plasma sample at 0.0300 mg/L colistin E2 (LLOQ), 15 mg/L for the IS; d) A plasma sample from a volunteer at 3.5 h after administration of a single dose of 5 mg/kg colistin base activity.

Six plasma lots were free of interference from the analyte. Typical chromatograms of plasma samples are shown in Fig. 3.

#### Matrix effect and recovery

The mean values of the recovery and matrix effects on colistin E2 and the IS are shown in Table 2. The recovery was consistent at different concentrations and the CV values were lower than 15%. The relatively low recovery might have been caused by the adsorption of colistin E2 on the lab materials^10^. The matrix effect was evaluated in six lots of normal human plasma, a lot of hemolytic plasma, and a large amount of hyperlipidemic plasma (n = 3 for each lot). The matrix factor (MF) was calculated for each lot of matrices by calculating the ratio of the peak area in the presence of the matrix to the peak area in the absence of the matrix. The CV of the IS-normalized MF calculated from the eight matrices was much less than 15%, indicating that the matrix had no effect on the quantization of the analyte.

**Table 2:** Recovery and matrix effect of colistin E2 and IS.

Item	Colistin E2 concentration (mg/L)				IS concentration (mg/L)
	0.0900	0.240	12.0	24.0	15
Recovery (%)	44.2 ± 2.1	42.1 ± 1.3	43.0 ± 1.8	41.1 ± 1.3	44.8 ± 1.4
Matrix effect (%CV of the IS-normalized MF)	1.1	2.6	1.1	0.7	–

#### Calibration curve, LLOQ, precision and accuracy

Satisfactory coefficients of the standard curve (r^2^:0.9960–0.9991) were obtained for the plasma samples. A linear regression with a 1/x^2^ weight best fit the data. The LLOQ for plasma was 0.0300 mg/L for colistin E2. Intra- (n = 6) and inter-day (n = 18) precision and accuracy were lower than 15%, which are presented in Table 3.

**Table 3:** Intra-/inter-day accuracy (%CV) and precision (%RE) of colistin E2 and CMS E2 in quality control plasma samples (n = 18).

Colistin assay	LLOQ	Low QC	Middle QC1	Middle QC2	High QC
	0.0300	0.0900	0.240	12.0	24.0
Intra-assay					
%CV	5.1–12.7	3.1–6.3	2.7–5.7	2.0–2.2	0.7–1.7
%RE	− 5.7–9.3	− 6.8–2.0	− 7.9 to − 2.1	0.0–3.3	− 1.7–0.8
Inter-ASSAY					
%CV	10.8	6.0	5.0	2.4	1.6
%RE	− 0.3	− 3.4	− 5.8	0.8	− 0.4

#### Carryover and sample dilution studies

Following the injection of a sample with a high colistin E2 concentration, a carryover effect was detected because colistin E2 easily adhered to various laboratory materials^15^. Using the method described by Zhou et al.,^10^ 300 μl 90% ACN with 2.0% FA as the strong wash solution and 900 μl 10% ACN with 0.2% FA as the weak wash solution were used to wash away colistin E2 adhered to the UHPLC–MS/MS system to eliminate the carryover effect. No significant effect of carryover was observed, and dilution integrity was demonstrated in plasma samples without loss of accuracy and precision, as shown in Table 4.

**Table 4:** Dilution integrity of colistin E2 in human plasma (Mean ± SD, n = 6).

Matrix	Target	Dilution QC (mg/L)	Dilution factor	%RE	%CV
		120			
Plasma	colistin E2	123 ± 1.79	5	1.5	1.5
	CMS E2	121 ± 1.72	5	0.8	1.4

#### Stability studies

The stability of colistin E2 was evaluated at two levels (low and high QCs). There was no degradation in stock solutions stored at ambient for 21 h and − 20 ℃ for at least 97 days in long-term stability studies. Furthermore, no degradation was observed under the following conditions: on the bench top in ice water for 24 h, four freeze–thaw cycles, storage of colistin E2-spiked samples at − 20 ℃ and − 80 ℃ (at least 93 days), and storage of extracts at 4 ℃ in an auto-sampler for 65.5 h. The stability of colistin E2 in fresh whole blood was also evaluated, and no degradation was observed in the blood on the bench top in ice water for 66 min, which covered the sampling procedure.

The hydrolysis of CMS E2 is temperature dependent; the lower the temperature, the slower the hydrolysis rate. The samples were processed in ice water. Therefore, we studied their stability in ice-water. In the presence of CMS E2 (the spiked concentration was 50 times the concentration of colistin, which can cover the clinical samples), the stability of colistin E2 was also evaluated under the following conditions: on the bench top in ice water for 21 h, four freeze–thaw cycles, storage at − 20 ℃ and − 80 ℃ (at least 98 days), and storage of extracts at 4 ℃ in an auto-sampler for 22.5 h. The stability of colistin E2 in blood spiked with CMS E2 was also evaluated, and the percentage of change was less than 15% under the following conditions: ice water for 60 min. As shown in Table 5, the stability of colistin E2 was not affected by storage or pretreatment in the presence of CMS E2.

**Table 5:** The stability result of colistin E2 in the matrix spiked with CMS E2.

Item		%Change
Plasma		
Benchtop stability	21 h in ice water	2.0
Freeze/thaw stability	Freeze/Thaw 4 times by − 20℃/ice water	− 2.7
	Freeze/Thaw 4 times by − 80℃/ice water	− 2.0
Long-term stability	41 d in freezer ( − 20℃)	1.3
	98 d in freezer ( − 20℃)	− 7.0
	41 d in freezer ( − 80 ℃)	− 1.3
	98 d in freezer ( − 80 ℃)	− 12.6
Blood		
Benchtop stability	60 min in ice water	− 3.1
Sample extracts		
Processed sample stability	22.5 h in an auto-sampler (4℃)	4.4

### Application of the assay in a clinical pharmacokinetics study

**Figure 4:**
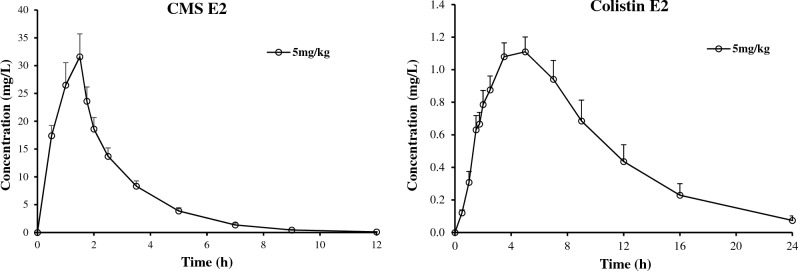
Mean Plasma concentration–time profiles following a single intravenous (5 mg colistin E2 base activity per kg) of CMS E2 to healthy Chinese volunteers (n = 8).

After completion of method validation, the UHPLC-MS/MS method was applied to analyze human plasma samples obtained from eight healthy Chinese volunteers in a pharmacokinetic study. The pharmacokinetic study was approved by the ethics committee of Huashan Hospital (Fudan University). Informed consent was obtained from all the study volunteers. After receiving a single intravenous of CMS E2 (dose of 5 mg/kg, given as colistin E2 base activity), blood samples of 4 ml were collected each time at 0 h (pre-dose), 30 min, 60 min, 1.5 h (end of infusion), 1.75, 2, 2.5, 3.5, 5, 7, 9, 12, 16, 24, 36, 48 h into labeled vacutainer collection tubes containing EDTA-K_2_ as an anticoagulant. All sample tubes were centrifuged at 0℃ ~ 2℃ for 10 min at 3500 rpm and the collected plasma samples were stored at -90℃ ~ -60℃. The samples were then processed and analyzed using the validated method. Incurred sample reanalysis was conducted with the following results: 100.0% of the repeated sample results were within 20% variability. The concentration-versus-time curves for colistin E2 and CMS E2 in a healthy subject are presented in Fig. 4. Compared with previously reported methods, our simple pretreatment method removed CMS E2 and successfully prolonged the stability of colistin E2 to at least 22.5 h in the auto-sampler (approximately 4 times longer than the previous methods^16,17^), which significantly increased the efficiency and productivity of the assay. The use of ^15^N-labeled colistin E2 as an IS also clearly eliminated the interference of polymyxin B mixtures or polymyxin B1.

## Conclusions

In this study, the UHPLC–MS/MS method enabled a more accurate quantification of free colistin E2 and CMS E2 in human plasma. The rapid and simple pretreatment procedure (120 min) and reduced analysis time (5 min) allowed 96 plasma samples to be assayed in 10 h. Furthermore, our method prevented the potential interference of IS with the analytes. Given the accuracy and speed of the high-throughput assay, our methodology has significant potential in large clinical pharmacokinetic studies, which has laid a solid foundation for the subsequent listing of CMS E2 and also provided technical guidance for the study of the pharmacokinetics of other antibiotics.

### Supplementary Information

Supplementary Information.
