# Supplementary material for: A quantitative method for determination of colistin E2 methanesulphonate in human plasma by 15N-labeled colistin E2
Source: Sci Rep. 2023 Oct 26;13:18348. doi: 10.1038/s41598-023-45256-3 (PMC10603063; doi:10.1038/s41598-023-45256-3)
Supplement: Supplementary file 1 — Supplementary Information. [file 41598_2023_45256_MOESM1_ESM.docx]

## A quantitative method for determination of colistin E2 methanesulphonate in human plasma by ^15^N-labeled colistin E2

1. Instruments and Materials

The following instruments and materials were applied to this method.

| Name | Manufacturer | Specifications and models |
| --- | --- | --- |
| Column | Phenomenex | Kinetex 2.6 μm, XB-C18, 100A, (50*2.1 mm) |
| LC-MS/MS | Sciex | Sciex Exion LC-Sciex Triple quad 5500 |
| Pipette | Eppendorf | 0.5-10 μL |
|  |  | 2-20 μL |
|  |  | 10-100 μL |
|  |  | 20-200 μL |
|  |  | 100-1000 μL |
|  |  | Electronic dispenser |
|  | Rainin | 10-100 μL LTS |
|  |  | 20-200 μL LTS |
|  |  | 100-1 mL LTS |
| Multichannel adjustable pipette | Rainin | 20-300 μL LTS |
| Multichannel pipette | Rainin | L8-300XLS |
| Multichannel pipette | Eppendorf | 30-300 μL |
| Ultrapure water machine | Millipore | Advantage A10 |
| Ultrasonic cleaning machine | Kunshan Hechuang Ultrasound Instrument Co., Ltd | KH-300DE |
|  |  | KQ-300E |
| Nitrogen blowing instrument | Stuart | F7210\SBHCONC\SBH130D |
| 96-well nitrogen blowing instrument | Hangzhou Miou Instrument Co., Ltd | NKD200-1A |
|  | Biotage | Turbo-vap 96 |
| Electronic balances | METTLER TOLEDO | XPE105 |
|  |  | XPE26 |
| Vortex oscillator | Shanghai Qingpu Huxi Instrument Factory | XW-80A |
|  | Nanjing Maipu Instrument Co., Ltd | Vortexgenie2 |
|  | IKA | Vortex 3 |
|  | DLAB | MX-S |
| Microplate thermostatic oscillator | Hangzhou Miou Instrument Co., Ltd | ST70-2 |
| Multi-tube vortex oscillator |  | DMT-2500 |
|  | VWR | VX-2500 |
| High-speed refrigerated centrifuges | Eppendorf | 5810R |
| Cooler | Thermo Scientific | REL1204V |
|  |  | UGL2320V |
| Ultra-low temperature freezer | Haier Group Corporation | DW-86L728J |
|  | Thermo | 88400V |
| Biosafety cabinets | Suzhou Antai Air Technology Co., Ltd | BSC-1004IIA2 |
|  |  | BSC-1304IIA2 |
| Graduated cylinder | KIMBLE | 1000 mL |
|  |  | 500 mL |
|  |  | 250 mL |
|  |  | 50 mL |
|  |  | 100 mL |
| Oasis WCX 96-well Plate | Waters | 30mg/96-well |
| Positive pressure device | Waters | 186006961 |

2. Method

The volume and weight used to prepare the solution can be adjusted proportionally.

2.1 Solution and matrix preparation

2.1.1 Solution 1: 5% ammonia solution

2 mL 30% ammonia was added to 10 mL water, and then mixed well.

2.1.2 Solution 2: acetonitrile/water (3: 7, containing 2% ammonia) solution

5 mL 30% ammonia, 22.5 mL acetonitrile and 47.5 mL water were mixed well.

2.1.3 Solution 3: 0.5% formic acid aqueous solution

0.5 mL formic acid was added to 99.5 mL water, and then mixed well.

2.1.4 Solution 4: 6% formic acid aqueous solution

6 mL formic acid was added to 94 mL water, and then mixed well.

2.1.5 Eluate: acetonitrile/water (3: 7, containing 6% formic acid)

30 mL acetonitrile, 6 mL formic acid and 64 mL water were mixed well.

2.1.6 Mobile phase A: 0.1% formic acid aqueous solution

1 mL formic acid was added to 999 mL water, and then mixed well.

2.1.7 Mobile phase B: methanol: acetonitrile (1: 1)

500 mL acetonitrile and 500 mL methanol were mixed well.

2.1.8 2% hemolytic plasma

Blank whole blood (anticoagulant EDTA-K2) was placed at -20°C to break all blood cells. 2 mL was added to 98 mL of blank plasma (anticoagulant EDTA-K2), and then mixed well.

2.1.9 High-fat plasma (containing fat emulsion 300 mg/dL)

Appropriate volume of the structural fat emulsion injection was added to the blank plasma (anticoagulant EDTA-K2), and mixed well to prepare high-fat plasma with a concentration of 300 mg/dL.

2.1.10 Methanol: water (1: 1)

100 mL methanol was mixed with 100 mL water.

2.1.11 Needle wash solution 01 (Port): methanol: acetonitrile: isopropanol: water: formic acid = 3: 3: 3: 1: 0.2

300 mL methanol, 300 mL acetonitrile, 300 mL isopropanol and 2.0 mL formic acid were added to 100 mL water, then mixed well and stored at room temperature.

2.1.12 Needle wash solution 02 (Pump): isopropanol aqueous solution (1: 9)

10 mL isopropanol was added to 90 mL water, and then mixed well.

2.1.13 2 mol/L sodium hydroxide solution

1.6 g sodium hydroxide was dissolved in 20 mL water.

2.1.14 1 mol/L sulfuric acid solution

27.8 mL sulfuric acid (98%) was dropped into 472.2 mL water in a breaker, gently stirred with a glass stick.

2.1.15 Methanol: water (9: 1)

900 mL methanol was mixed with 100 mL water.

2.2 Preparation of internal standard solutions

Both IS-A and IS-D were kept at -20°C.

2.2.1 Internal standard stock solution A (IS-A)

The isotope-labeled colistin E2 was precisely weighed, dissolved with an appropriate amount of methanol: water (1: 1), and prepared IS-A with a concentration of 1.5 mg/mL.

2.2.2 Internal standard working solution D (IS-D)

100 μL IS-A was added to 9.9 mL of methanol: water (1: 1), and mixed well to prepare 15 μg/mL IS-D.

2.3 Preparation of colistin E2 stock and working solution

The standard curve stock solution and the quality control stock solution for colistin E2 need to be prepared from different weighing. All solutions were kept at -20°C.

2.3.1 Colistin E2 Stock Solution A (SS-A)

The colistin E2 reference was accurately weighed and dissolved with an appropriate amount of methanol: water (1: 1) to prepare a stock solution SS-A with a concentration of 3 mg/mL.

2.3.2 Colistin E2 Stock Solution B (SS-B)

1.2 mL SS-A was added to 1.8 mL of methanol: water (1: 1) to prepare a 1200 μg/mL stock solution SS-B.

2.3.3 Colistin E2 Stock Solution C (SS-C)

100 μL SS-B was added to 1.9 mL of methanol: water (1:1) to prepare a 60 μg/mL stock solution SS-C.

2.3.4 Colistin E2 standard curve and quality control working solution

Quality control working solution was prepared using methanol: water (1: 1) according to the following table:

| Working solution | Source solution | | | solvent | Working solution | |
| --- | --- | --- | --- | --- | --- | --- |
| Name | Name | Colistin E2 concentration (μg/mL) | Volume  (μL) | Volume  (μL) | Volume  (μL) | Colistin E2 concentration (μg/mL) |
| W-STD_8 | SS-B1 | 1200 | 500 | 500 | 1000 | 600 |
| W-STD_7 | SS-B1 | 1200 | 450 | 550 | 1000 | 540 |
| W-STD_6 | SS-B1 | 1200 | 250 | 750 | 1000 | 300 |
| W-STD_5 | SS-B1 | 1200 | 125 | 875 | 1000 | 150 |
| W-STD_4 | SS-C1 | 60 | 500 | 500 | 1000 | 30 |
| W-STD_3 | SS-C1 | 60 | 100 | 900 | 1000 | 6 |
| W-STD_2 | SS-C1 | 60 | 20 | 980 | 1000 | 1.2 |
| W-STD_1 | SS-C1 | 60 | 10 | 990 | 1000 | 0.6 |
| W-QCD | SS-A2 | 3000 | 800 | 200 | 1000 | 2400 |
| W-QCH | SS-B2 | 1200 | 400 | 600 | 1000 | 480 |
| W-QCM2 | SS-B2 | 1200 | 200 | 800 | 1000 | 240 |
| W-QCM1 | SS-C2 | 60 | 80 | 920 | 1000 | 4.8 |
| W-QCL | SS-C2 | 60 | 30 | 970 | 1000 | 1.8 |

Note: W-STDs and W-QCs (including W-LLOQ and W-QCD) are formulated from different SSs.

2.3.5 Colistin E2 standard curve and plasmatic samples

The standard curve of colistin E2 and quality control plasma samples were prepared according to the table below, and then stored at -20°C:

| Working solution | | | Standard curve and quality controlled plasma samples | | | |
| --- | --- | --- | --- | --- | --- | --- |
| Name | Colistin E2 (μg/mL) | Volume  (µL) | Human plasma volume (µL) | Total volume (µL) | Colistin E2 (μg/mL) | Name |
|  |  |  |  |  |  |  |
| W-STD_8 | 600 | 50 | 950 | 1000 | 30 | STD_8 |
| W-STD_7 | 540 | 50 | 950 | 1000 | 27 | STD_7 |
| W-STD_6 | 300 | 50 | 950 | 1000 | 15 | STD_6 |
| W-STD_5 | 150 | 50 | 950 | 1000 | 7.5 | STD_5 |
| W-STD_4 | 30 | 50 | 950 | 1000 | 1.5 | STD_4 |
| W-STD_3 | 6 | 50 | 950 | 1000 | 0.3 | STD_3 |
| W-STD_2 | 1.2 | 50 | 950 | 1000 | 0.06 | STD_2 |
| W-STD_1 | 0.6 | 50 | 950 | 1000 | 0.03 | STD_1 |
| W-QCD | 2400 | 50 | 950 | 1000 | 120 | QCD |
| W-QCH | 480 | 50 | 950 | 1000 | 24 | QCH |
| W-QCM2 | 240 | 50 | 950 | 1000 | 12 | QCM2 |
| W-QCM1 | 4.8 | 50 | 950 | 1000 | 0.24 | QCM1 |
| W-QCL | 1.8 | 50 | 950 | 1000 | 0.09 | QCL |

2.3.6 Colistin E2 diluted quality control samples

Matrix dilution (5-fold): 50 μL of QCD sample was mixed with 200 μL of blank human plasma and then extracted.

2.4 Preparation of CMS E2 stock solution and working solution

The quality control stock solution of CMS E2 should be stored at -20 °C, and the concentration of all CMS E2 solutions should be calculated as hydrolyzed free colistin E2.

2.4.1 CMS E2 stock solution A (SS-A_CMS E2)

CMS E2 reference was accurately weighed and dissolved with an appropriate amount of methanol: water (9: 1) to prepare a stock solution SS-A_CMS E2 with a concentration of 2 mg/mL (the correction factor should be calculated as free colistin E2).

2.4.2 Colistin E2 stock solution B (SS-B_CMS E2)

1.2 mL SS-A was added to 0.8 mL of methanol: water (9: 1) and mixed to prepare 1200 μg/mL stock solution SS-B_CMS E2.

2.4.3 Colistin E2 stock solution C (SS-C_CMS E2)

100 μL SS-B was added to 1.9 mL of methanol: water (9: 1) and mixed to prepare a 60 μg/mL stock solution SS C_CMS E2.

2.4.4 CMS E2 quality control working solution

The working solution for quality control was prepared using methanol: water (9: 1) according to the following table:

| Working solution | Source solution | | | solvent | Working solution | |
| --- | --- | --- | --- | --- | --- | --- |
| Name | Name | CMS E2 concentration (μg/mL) | Volume  (μL) | Volume  (μL) | Volume  (μL) | CMS E2 concentration (μg/mL) |
| W-QCD_CMS E2 | SS-A | 2000 | 600 | 400 | 1000 | 1200 |
| W-QCH_CMS E2 | SS-B | 1200 | 200 | 800 | 1000 | 240 |
| W-QCM2_CMS E2 | SS-B | 1200 | 100 | 900 | 1000 | 120 |
| W-QCM1_CMS E2 | SS-C | 60 | 80 | 920 | 1000 | 4.8 |
| W-QCL_CMS E2 | SS-C | 60 | 30 | 970 | 1000 | 1.8 |

2.4.5 CMS E2 standard curve and quality control plasma samples

Quality control plasma samples of CMS E2 were prepared according to the table below, and stored at -20°C (all plasma samples prepared with CMS E2 were measured at free colistin E2):

| Working solution | | | Standard curve and quality controlled plasma samples | | | |
| --- | --- | --- | --- | --- | --- | --- |
| Name | CMS E2 (μg/mL) | Name | CMS E2 (μg/mL) | Name | CMS E2 (μg/mL) | Name |
|  |  |  |  |  |  |  |
| W-QCD_CMS E2 | 1200 | 50 | 950 | 1000 | 60 | QCD_CMS E2 |
| W-QCH_CMS E2 | 240 | 50 | 950 | 1000 | 12 | QCH_CMS E2 |
| W-QCM2_CMS E2 | 120 | 50 | 950 | 1000 | 6 | QCM2_CMS E2 |
| W-QCM1_CMS E2 | 4.8 | 50 | 950 | 1000 | 0.24 | QCM1_CMS E2 |
| W-QCL_CMS E2 | 1.8 | 50 | 950 | 1000 | 0.09 | QCL_CMS E2 |

2.4.6 CMS E2 diluted quality control samples

Matrix dilution (5-fold): 50 μL of QCD_CMS E2 sample was mixed with 200 μL of blank human plasma and then extracted.

2.5 System Adaptability Sample (SVS)

One sample or solution should be prepared at the appropriate concentration (LLOQ concentration level) for use as a system-adapted sample.

2.6 Preparation of quality control samples containing CMS E2/colistin E2

The method was used for the stability study, and all the following medicinal solutions and plasma samples were prepared and used in an ice water bath and stored at -20 °C after use.

2.6.1 CMS E2 stock solution A (SS-A_CMS E2)

CMS E2 reference was accurately weighed and dissolved with an appropriate amount of methanol: water (9: 1) to prepare a stock solution SS-A_CMS E2 with a concentration of 2 mg/mL (the correction factor should be calculated as free colistin E2).

2.6.2 CMS E2 working solution (W_CMS E2)

0.96 mL SS-A was added to 1.04 mL of methanol: water (9: 1) and mixed well to make a 960 g/mL working solution W_CMS E2.

2.6.3 Colistin E2 working solution (W_E2)

96 μL W-QCM2 was added to 1.104 mL of methanol: water (1: 1) and mixed well to prepare a 19.2 μg/mL working solution W_E2.

2.6.4 Preparation of plasma quality control sample QC_mix for CMS E2/Colistin E2

| Working solution | | | Standard curve and quality controlled plasma samples | | | |
| --- | --- | --- | --- | --- | --- | --- |
| Name | CMS E2/ Colistin E2 (μg/mL) | Volume  (µL) | Human plasma  Volume (µL) | Total volume  (µL) | CMS E2/ Colistin E2 (μg/mL) | Name |
|  |  |  |  |  |  |  |
| W_CMS E2/ W_E2 | 960/19.2 | 10/10 | 380 | 400 | 24/0.48 | QC_mix |

2.7 Instrument Conditions

The followings are the mass spectrometry conditions.

2.7.1 Mass spectrometry systems

Mass spectrometer: Sciex 5500 triple quadrupole mass spectrometer

Ionization mode: (+) ESI

Scan Mode: Multi-Reaction Monitoring Mode (MRM)

| Parameter | Optimize the value |
| --- | --- |
| Ion Spray (IS) | 5500 |
| Curtain Gas (CUR) | 30 |
| Temperature (TEM) | 550 |
| Entrance Potential (EP) | 10 |
| Collision Gas (CAD) | 9 |
| Collision Cell Exit Potential (CXP) | 10 |
| Dwell Time (ms) | 100 |
| Gas 1 | 40 |
| Gas 2 | 40 |

2.7.2 Precursor ions, fragment ions, declustering voltage (DP) and collision energy (CE)

| Compound | Precursor ions (*m/z*) | fragment ions (*m/z*) | CE (eV) | DP |
| --- | --- | --- | --- | --- |
| Colistin E2 | 386.2 | 101.1 | 20 | 120 |
| IS | 391.4 | 103.1 | 20 | 120 |

3 Atlas

*Figure S1*. Lower limit of colistin E2 quantification in human plasma (LLOQ, 0.03 g/mL) chromatogram (A: colistin E2 and B: IS)


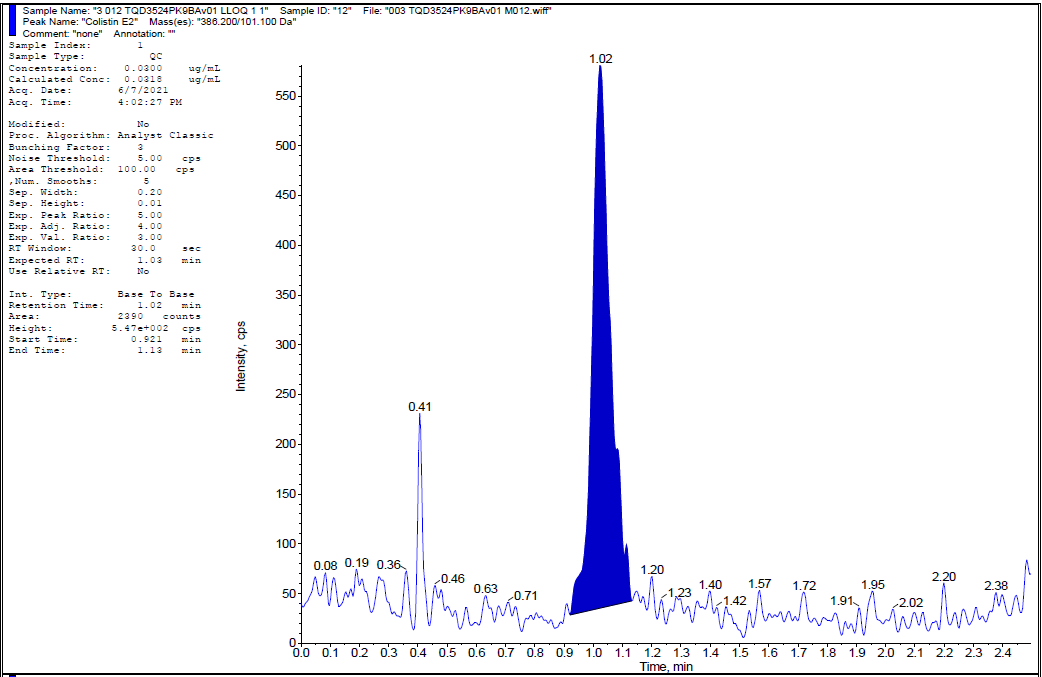

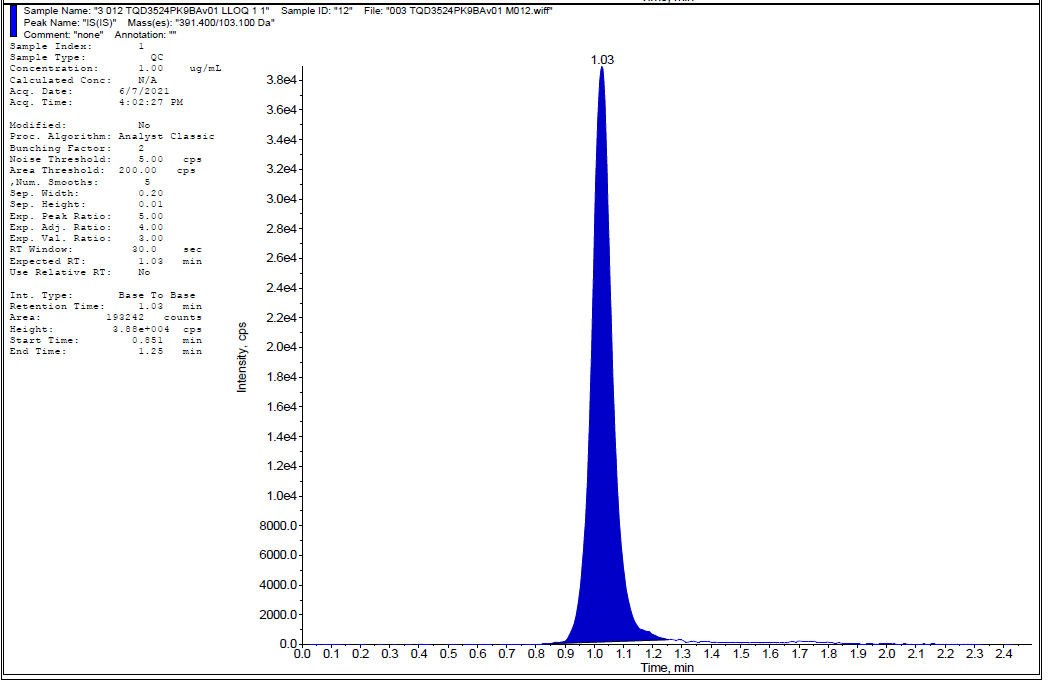


(B)

(A)

*Figure S2*. Low-concentration plasma-controlled plasma samples (QCL, 0.09 g/mL) from human plasma with CMS E2 hydrolyzed by sulfuric acid (A: colistin E2 and B: IS)


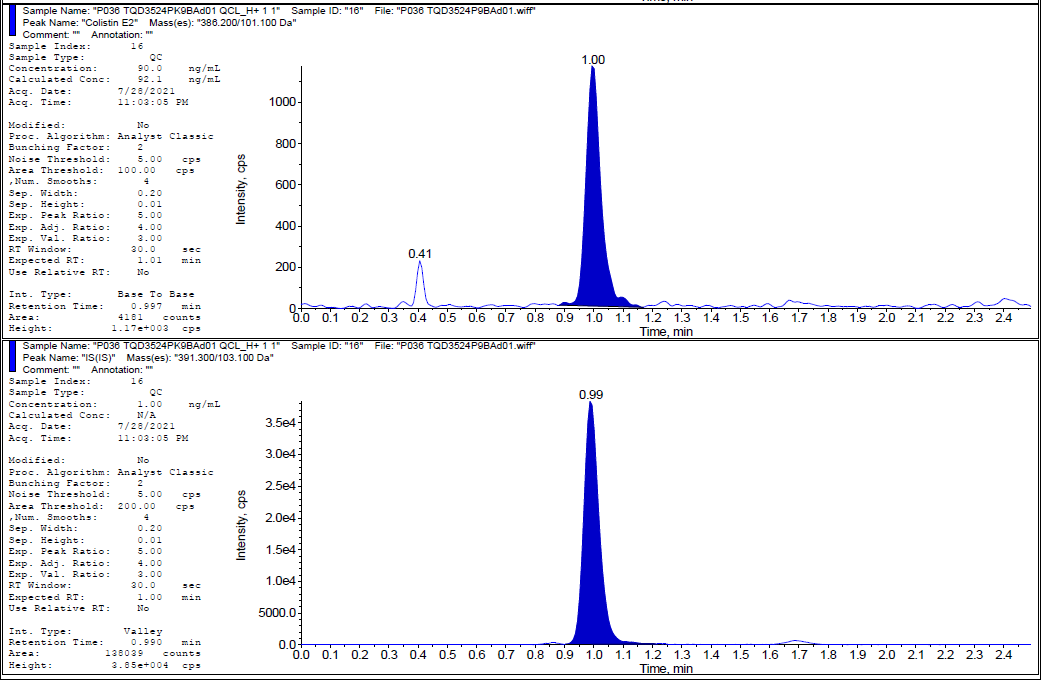


*Figure S3*. Full scan (A) and fragment ion scan (B) of colistin E2





[M+3H]^3+^

(B)

(A)

*Figure S4*. Full scan (A) and fragment ion scan (B) of IS





[M+3H]^3+^

(A)

(B)
